# Analysis of Herpes Simplex Virus Reactivation in Explant Reveals a Method-Dependent Difference in Measured Timing of Reactivation

**DOI:** 10.1128/JVI.00848-17

**Published:** 2017-07-27

**Authors:** Jessica R. Doll, Nancy M. Sawtell

**Affiliations:** a Cincinnati Children's Hospital Medical Center, Division of Infectious Diseases, Cincinnati, Ohio, USA; b University of Cincinnati, Department of Molecular Genetics, Biochemistry, and Microbiology, Cincinnati, Ohio, USA; University of California, Irvine

**Keywords:** explant, herpes simplex virus, latency, methodology, reactivation

## Abstract

Herpes simplex virus (HSV) infection is widespread in the human population. Following orofacial infection, HSV establishes latency in innervating sensory neurons, primarily located in the trigeminal ganglia. A central feature of HSV pathogenesis is the ability to periodically reactivate in those neurons and be transported back to the body surface. Both transmission and disease, such as keratitis, encephalitis, and neurodegeneration, have been linked to reactivation. Despite invaluable insights obtained from model systems, interactions between viral and host functions that regulate reactivation are still incompletely understood. Various assays are used for measuring reactivation in animal models, but there have been limited comparisons between methods and the accuracy of detecting the timing of reactivation and the corresponding amount of infectious virus produced in the ganglia per reactivation event. Here, we directly compare two approaches for measuring reactivation in latently infected explanted ganglia by sampling media from the explanted cultures or by homogenization of the ganglia and compare the results to viral protein expression in the whole ganglia. We show that infectious virus detection by direct homogenization of explanted ganglia correlates with viral protein expression, but detection of infectious virus in medium samples from explanted cultures does not occur until extensive spread of virus is observed in the ganglia. The medium-sampling method is therefore not reflective of the initial timing of reactivation, and the additional variables influencing spread of virus in the ganglia should be considered when interpreting results obtained using this method.

**IMPORTANCE** The development of treatments to prevent and/or treat HSV infection rely upon understanding viral and host factors that influence reactivation. Progress is dependent on experimental methods that accurately measure the frequency and timing of reactivation in latently infected neurons. In this study, two methods for detecting reactivation using the explant model are compared. We show through direct tissue homogenization that reactivation occurs much earlier than can be detected by the indirect method of sampling media from explanted cultures. Thus, the sampling method does not detect the initial timing of reactivation, and results obtained using this method are subject to additional variables with the potential to obscure reactivation outcomes.

## INTRODUCTION

Herpes simplex virus (HSV) is transmitted through close contact at the body surface, and viral replication results in spread to innervating sensory neurons during acute infection. The virus can then enter a latent state in neurons of the peripheral and central nervous systems. Periodically, HSV reactivates from this latent state and produces infectious virus that is transported back to the body surface and can be transmitted to a new host (1). These reactivation events can also result in accumulating tissue damage, such as keratitis (2), encephalitis (3), and neurodegeneration (4–6).

Stevens and colleagues provided the first direct evidence that latent HSV reactivated from sensory ganglia. Ganglia axotomized from latently infected mice were shown to be negative for infectious virus; however, when cultured for up to 30 days, infectious virus was detected (7–9). Several variations of explant reactivation have been employed since this approach was originally developed. Although not comprehensive, some examples of methods derived from this protocol are (i) maintenance of the whole ganglia in culture and homogenization of the ganglia at a given time postexplant (PE) to assay for infectious virus directly (10–15), (ii) daily sampling of the culture medium until infectious virus is detected (16, 17), (iii) dividing the ganglia into small pieces and maintaining them on indicator cell monolayers until infectious virus is detected (18), and (iv) enzymatic dissociation of ganglia and maintenance of the dissociated cells as monolayers until infectious virus is detected (19–22). These different approaches to studying explant-induced reactivation have been used by various groups, but the impact of the methodology on the experimental results and conclusions has received little attention. It is reasonable to assume that if reactivation-competent virus is present in the ganglia and the ganglia are maintained in culture, it will eventually be detected by any of the explant methods described. However, the validity of these assays to characterize more detailed parameters, such as the initial timing of reactivation, has been underinvestigated.

Infectious virus has been detected as early as 14 h postexplant (12) and consistently at 22 to 24 h postexplant (10, 11, 15) when the latently infected, explanted ganglia are homogenized and assayed directly by plaque assay. However, when medium samples from the explanted cultures are tested, infectious virus has rarely been detected prior to 4 days postexplant (16, 17). This large time difference raised the possibility that the sampling method does not accurately measure the initial time of reactivation. To test this, a direct comparison of the timing of initial primary reactivation, as determined by the detection of infectious virus, by either daily sampling of explanted culture media or direct homogenization of explanted ganglia was performed. Because this was done in trigeminal ganglia (TG) from the same groups of latently infected mice, differences observed in the time that reactivation was first detected could be attributed to the methods utilized. In addition, representative ganglia were examined throughout the explant time period (up to 144 h postexplant) to identify the extent and cellular distribution of viral protein expression.

Here, we report a dramatic difference in the timing of detection of infectious virus by these two methods. In the same TG, homogenization revealed that 93% contained infectious virus within 48 h postexplant, and thus, reactivation had already occurred by that time. In contrast, the sampled-medium approach registered 99% of these ganglia as reactivation negative. Similar results were found in latently infected, explanted TG from susceptible (Swiss Webster) mice and resistant (C57BL/6) mice. Our results demonstrate that while sampling of media from explanted cultures allows monitoring of the same ganglia over time, the outcome does not accurately reveal the timing of initial reactivation in explant, and thus, the method is not suitable for this application.

## RESULTS

### Initial primary reactivation in explanted TG occurred 3 days prior to detection of infectious virus in the culture medium.

A comparison of two detection methods to measure the time of HSV reactivation in explant was performed by (i) daily sampling of media from explanted cultures and (ii) homogenization of the ganglion and plating on rabbit skin cells (RSC). The results of these methods were also compared to viral protein expression in the ganglia. Forty male Swiss Webster mice were infected on scarified corneas with 2 × 10^5^ PFU of HSV-1 strain 17syn+. Six mice succumbed to acute infection (15% mortality). Following the establishment of latency (>45 days postinfection), the TG pair from each of the remaining 34 animals was removed, and each TG was individually explanted into the wells of a 24-well plate as detailed in Materials and Methods. Medium samples (100 μl) were collected from each ganglion culture at 24-h intervals and directly plated onto RSC monolayers. At each 24-h time point, a subset of ganglia were homogenized and assayed for infectious virus as reported previously (11). Additional ganglia at each 24-h interval were examined by whole-ganglion immunohistochemistry for viral proteins (23) to determine the number of neurons that had entered the lytic program and the extent and timing of viral spread within the tissue.

**FIG 1:**
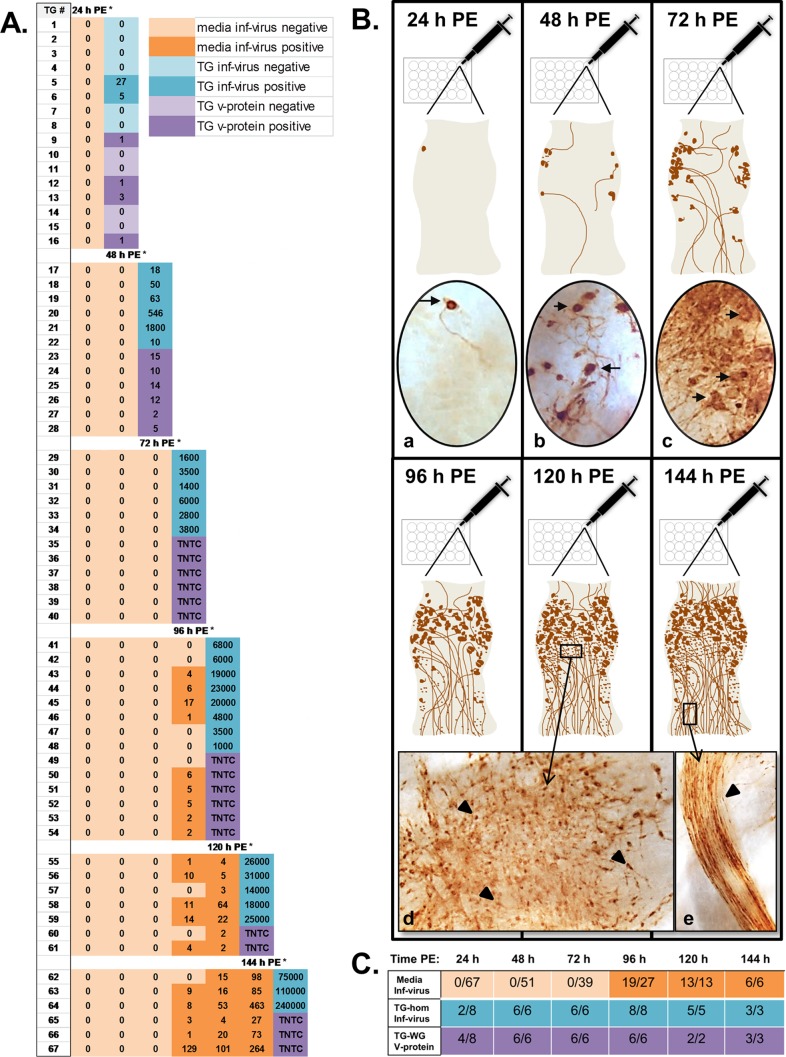
Comparison of timing of reactivation in explant of 17syn+ from latently infected TG using medium sampling and tissue homogenization. Latently infected (17syn+) trigeminal ganglia were aseptically removed from Swiss Webster mice and individually explanted in 1.5 ml medium. One hundred microliters of medium from each TG was collected daily, directly plated onto RSC monolayers in individual wells of a 24-well plate, and overlaid with 1% carboxymethylcellulose (CMC). (A) The number of plaques (PFU per 100 μl) detected for each sample from each TG from 24 to 144 h PE is shown (orange boxes). In addition, at each sampling point, a subset of TG was homogenized, and infectious virus was quantified by standard plaque assay (numbers in blue boxes; PFU/100 μl). *, a Mann-Whitney test comparing titers recovered in sampled media and homogenized tissue showed significant differences (*P* < 0.05). Additional TG were collected at each sampling point and processed for whole-ganglion immunohistochemistry (IHC) to detect viral protein as an additional measure of the transition from the latent into the lytic cycle (number of neurons expressing viral proteins per ganglion; purple boxes). Beyond 48 h PE, the neurons were too numerous to count (TNTC) (cf. panel B, c). (B) Pictorial summary of viral protein expression at increasing times PE. The photomicrographs of representative TG show that the number of neurons expressing viral proteins increased steadily from 24 h to 48 h PE (a and b) and paralleled detection of infectious virus in the homogenized tissue, but not in sampled media. Spread to neighboring cells and along the axons was observed within 48 h PE and became extensive by 72 h and thereafter. (d and e) Involvement of nonneuronal cells (arrowheads) at 96 h PE (d) and axon bundles (e) marked by viral proteins as time in explant increased. Importantly, detection of infectious virus in the medium samples was first detected only at this time and at very low levels. The dark-brown areas are immunoperoxidase reaction products marking the presence of HSV proteins. (C) Summary table of data from panel A showing the number of positive ganglia out of the total ganglia tested at each time point for each method.

Variation among the individual ganglia was anticipated. To eliminate this variation as a component of any differences observed between homogenization and medium sampling, the 100-μl medium samples and final homogenization or fixation were tracked for each individual ganglion for the period examined (Fig. 1A). Infectious virus was not detected in TG homogenized directly postdissection (0/8); however, within 24 h PE, reactivation was detected in 25% (2/8) of homogenized ganglia (range, 5 to 27 PFU/100 μl), and 50% (4/8) of the ganglia contained neurons (range, 1 to 3 neurons/ganglion) expressing viral proteins (Fig. 1A and C). As reported previously, viral protein was restricted to individual neurons at this early time (11). By 48 h PE, 100% (6/6) of the ganglia contained infectious virus (range, 10 to 1,800 PFU/100 μl) and 100% (6/6) of the ganglia were positive for viral protein expression (range, 2 to 15 neurons/ganglion). Viral proteins were also observed in support cells and along axons at 48 h PE (Fig. 1B, b), evidence that virus had already spread beyond the sites of primary reactivation at that time. In striking contrast, reactivation was not detected in 100-μl samples of media at 24 h (0/67), 48 h (0/51), or 72 h (0/39) PE, even though at this later time, 1,400 to 6,000 PFU/100 μl were recovered from 100% (6/6) of ganglia following homogenization (Fig. 1A and C). Consistent with this finding, extensive viral spread was observed in 100% (6/6) of the ganglia processed for viral protein expression at 72 h PE (Fig. 1B, c). Paralleling a previous study (17), at 96 h PE, 70% (19/27) of the 100-μl medium samples were positive for infectious virus, but only 1 to 129 PFU/100 μl was recovered compared to 1,000 to 23,000 PFU/100 μl recovered from 100% (8/8) of the homogenized ganglia at this time. Additionally, continued spread of viral proteins to nonneuronal cells and along the axons was observed in 100% (6/6) of ganglia at 96 h PE (Fig. 1B, d and e). These results demonstrate that in these ganglia, primary reactivation occurred within the first 48 h PE. The medium-sampling method failed to detect reactivation until extensive viral spread had occurred within the ganglion. By 120 h PE, 100% (13/13) of the medium samples were positive for infectious virus (Fig. 1A and C); however, the average recovery was nearly 3 log units less than the average recovery in homogenized ganglia (24 ± 30 PFU/100 μl versus 22,800 ± 6,760 PFU/100 μl; Mann-Whitney test; *P* = 0.0002). Similarly, at the latest time point examined (144 h PE), the average recovery in medium samples was still 3 log units less than the average recovery in homogenized ganglia (168 ± 165 PFU/100 μl versus 141,667 ± 86,939 PFU/100 μl; Mann-Whitney test; *P* = 0.02) (Fig. 1A and C), emphasizing the lack of sensitivity of the sampling method for detection of not only initial reactivation, but also the presence of extensive spread and replication of infectious virus in the explanted tissue.

### Explant reactivation of latently infected TG from C57BL/6 mice parallels observations from Swiss Webster mice.

To test the possibility that there was a mouse strain-dependent effect on detection of reactivation in media from explanted cultures versus homogenized explanted tissue, 23 male C57BL/6 mice were infected with 2 × 10^6^ PFU of HSV-1 strain 17syn+. One mouse died during acute infection (4% mortality), and latently infected TG (>45 days postinfection) from the remaining 22 mice were explanted as described above. A subset of ganglia were homogenized for infectious virus or processed for immunohistochemistry at 24, 48, 72, or 96 h PE to compare to infectious virus detected in 100-μl medium samples taken at the same time.

Infectious virus was detected at 24 h PE in 25% (2/8) of homogenized ganglia (Table 1). By 48 h PE, 83% (5/6) of the ganglia had reactivated, and by 72 h PE, 100% (4/4) of the explanted TG contained infectious virus, as determined by homogenizing the tissue. In contrast, reactivation was not detected in 100-μl medium samples at 24 (0/44), 48 (0/28), or 72 (0/16) h PE. In agreement with the first experiment, infectious virus was first detected in 100-μl medium samples at 96 h PE (2/8); however, the amount of infectious virus recovered and the frequency of samples that were positive were significantly different from homogenized TG (Mann-Whitney test; *P* = 0.002) (Table 1).

**TABLE 1:** Explant reactivation of 17syn+ from latently infected C57BL/6 male mice^*a*^

Time (h) PE	No. positive/no. tested (%)		Recovered infectious titers (PFU/100 μl) (avg^*b*^ ± SD)		*P* value
	Medium	Tissue	Medium	Tissue	
24	0/44 (0)	2/8 (25)	ND^*c*^	4 ± 4	0.0211
48	0/28 (0)	5/6 (83)	ND	4.3 × 10^1^ ± 3.4 × 10^1^	<0.0001
72	0/16 (0)	4/4 (100)	ND	6.2 × 10^2^ ± 4.2 × 10^2^	0.0002
96	2/8 (25)	4/4 (100)	2 ± 1	1.6 × 10^3^ ± 2.6 × 10^3^	0.0020

a Trigeminal ganglia from C57BL/6 mice latently infected with HSV-1 strain 17syn+ were explanted in 1.5 ml medium, and 100-μl samples were removed daily. A subset of ganglia were homogenized in 1 ml medium at each time point. A Mann-Whitney test comparing the amounts of infectious virus detected in sampled media and homogenized tissue showed significant differences at every time point examined (*P* < 0.05).

b avg, average recovery in positive samples.

c ND, none detected.

**FIG 2:**
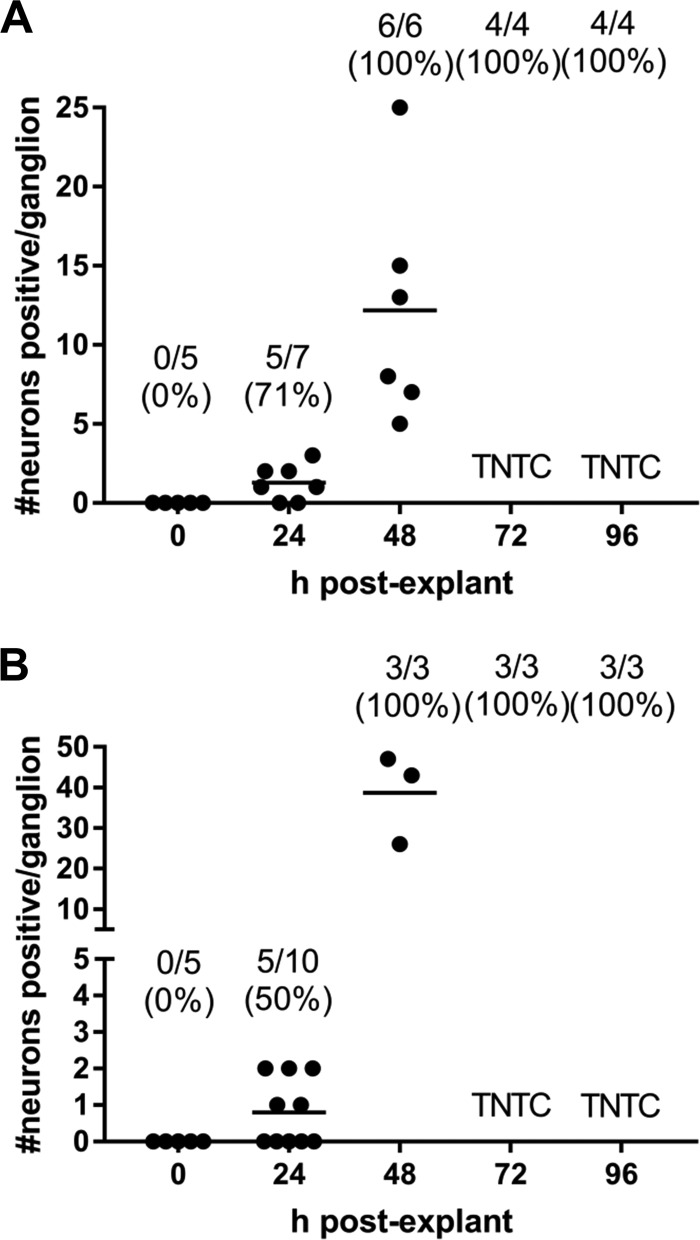
Viral protein detection in explanted TG from C57BL/6 mice. Latently infected trigeminal ganglia were aseptically removed from C57BL/6 mice and individually explanted in 1.5 ml medium. At each time point PE, a subset of ganglia was processed for whole-ganglion IHC, as described in Materials and Methods, and the neurons expressing viral proteins were counted. Each point represents an individual ganglion, and the line indicates the mean number of neurons positive per ganglion. The total number of ganglia positive out of the total number examined is given above each time point. After 48 h PE, there was extensive viral spread throughout the ganglia, and the neurons were too numerous to count (TNTC). (A) TG explanted from C57BL/6 male mice latently infected with 17syn+. (B) TG explanted from C57BL/6 female mice latently infected with McKrae.

Immunohistochemical analysis of explanted TG from C57BL/6 mice latently infected with 17syn+ showed a progression of viral protein expression and spread similar to that observed in the initial experiment with TG explanted from Swiss Webster mice. Individual neurons expressing viral proteins were detected at 24 h PE in 71% (5/7) of ganglia (1 to 3 neurons/ganglion) (Fig. 2A). Similar to the first experiment, by 48 h PE, there was spread of viral proteins beyond sites of primary reactivation (6/6 ganglia). Spread to nonneuronal cells became more extensive at 72 h (4/4 ganglia) and 96 h (4/4 ganglia) PE (Fig. 2A).

In addition to the mouse strain, the viral strain could influence the time at which infectious virus is detected in the medium compared to the whole homogenized ganglia. To test this possibility, explant reactivation of the highly virulent HSV-1 strain McKrae was examined. As reported previously (24, 25), high mortality (88%) was observed following infection of male mice with McKrae, so female mice were selected for this experiment. Thirty-six C57BL/6 female mice were infected with 1 × 10^5^ PFU of HSV-1 strain McKrae, and 15 died during acute infection (42% mortality). The experiment described above was performed with latently infected TG (>45 days postinfection) from the remaining 21 mice. Infectious virus was recovered from 3/7 (43%) homogenized ganglia at 24 h PE and 100% of ganglia at 48, 72, and 96 h PE (Table 2). Similarly to 17syn+ explanted ganglia, infectious virus was not detected in 100-μl medium samples at 24 h PE (0/42). Infectious virus was first detected in a single 100-μl medium sample at 48 h PE (1/25), and more medium samples were positive at 72 h PE (12/19). At 96 h PE, 100% of the media from the cultures were positive (13/13) (Table 2). As observed in the previous two experiments, the amount of infectious virus recovered and the frequency of samples that were positive were significantly different from homogenized TG (Mann-Whitney test *P* < 0.05) (Table 2). Immunohistochemical analysis revealed similar progressions of reactivation and spread with McKrae- and 17syn+-infected TG. Viral proteins were detected at 24 h PE in 50% (5/10) of ganglia (1 to 2 neurons/ganglion), and spread to nonneuronal cells was apparent by 48 h PE (3/3 ganglia) and increased at 72 (3/3 ganglia) and 96 (3/3 ganglia) h PE (Fig. 2B). Although the cells expressing viral proteins were too numerous to count at 72 and 96 h PE for both McKrae and 17syn+ explanted ganglia, more cells contained viral proteins by 48 h PE and spread was more extensive in the McKrae TG than in the explanted TG from 17syn+-infected mice (39 ± 11 neurons for McKrae versus 12 ± 7 neurons for 17syn+ at 48 h PE; Mann-Whitney test; *P* = 0.02) (Fig. 2). This is consistent with higher infectious virus titers recovered in homogenized TG (Table 2).

**TABLE 2:** Explant reactivation of McKrae from latently infected C57BL/6 mice^*a*^

Time (h) PE	No. positive/no. tested (%)		Recovered infectious titers (PFU/100 μl) (avg^*b*^ ± SD)		*P* value
	Medium	Tissue	Medium	Tissue	
24	0/42 (0)	3/7 (43)	ND^*c*^	4 ± 1	0.0019
48	1/25 (4)	3/3 (100)	8	8.6 × 10^2^ ± 5.7 × 10^2^	0.0003
72	12/19 (63)	3/3 (100)	41 ± 95	1.6 × 10^4^ ± 6.7 × 10^3^	0.0006
96	13/13 (100)	10/10 (100)	43 ± 59	4.1 × 10^4^ ± 4.0 × 10^4^	<0.0001

a Trigeminal ganglia from C57BL/6 mice latently infected with HSV-1 strain McKrae were explanted in 1.5 ml medium, and 100-μl samples were removed daily. A subset of ganglia were homogenized in 1 ml medium at each time point. A Mann-Whitney test comparing the amounts of infectious virus detected in sampled media and homogenized tissue showed significant differences at every time point examined (*P* < 0.05).

b avg, average recovery in positive samples.

c ND, none detected.

## DISCUSSION

The goal of our study was to compare the abilities of the explant medium-sampling method and direct tissue homogenization to accurately detect the time to reactivation occurring in explanted TG latently infected with HSV. To do this, the timing of reactivation was measured by (i) detection of infectious virus in medium samples or homogenized TG combined with (ii) spatiotemporal analysis of the transition from the absence of viral protein expression (latency) to protein expression in discrete neurons (exit from latency). Utilizing the same set of explanted TG, the results of these measures were directly compared.

We use the classic operational definition of reactivation, specifically, the detection of infectious virus following a latent state. In this study, the direct homogenization approach allowed detection of initial reactivation in the explanted ganglia within 24 to 48 h after placement of axotomized whole ganglia into culture; although it is possible that low levels of infectious virus could have been detected at an even earlier time postexplant (12). Importantly, the number of neurons that had exited latency at 24 h PE (1 to 3 neurons/ganglion) was consistent with the low level of infectious virus detected in homogenized ganglia (1 to 27 PFU/100 μl). These characteristics of initial primary reactivation (i.e., low levels of virus produced in rare, discrete neurons) (Fig. 1) are consistent with previous reports (11, 23, 26). It is notable that within 48 h PE, localization of virus within the TG is more complex, a mixture of primary reactivation events and secondary spread of virus into neurons and support cells (Fig. 1B, b). Indeed, the presence of infectious virus in the medium following reactivation would require a level of viral replication within the ganglia sufficient for escape of virus into the medium. This provides a reasonable explanation for the discrepancy in timing of reactivation based on detection of infectious virus within the TG or the detection of infectious virus in the medium sample. In this study, medium samples remained overwhelmingly negative until the viral titer in the TG achieved ∼100,000 PFU. Although this level of virus in the TG was associated with a high percentage (>60%) of positive medium samples, the titers in the media averaged only 28 PFU/100 μl, suggesting that escape of virus from the TG into the medium is inefficient.

Our findings suggest an additional point, namely, that the viral strain may influence replication and spread of virus downstream of the primary reactivation event. For example, we observed similar infectious virus titers (Mann-Whitney test; *P* = 0.5170) and numbers of neurons exiting latency (Mann-Whitney test; *P* = 0.4122) in HSV strain McKrae- and 17syn+-infected ganglia at 24 h PE (Tables 1 and 2 and Fig. 2). However, analysis of viral titers over the next 72 h demonstrated that the titers in McKrae-infected TG (linear regression; slope = 3,939 PFU/h; *R*^2^ = 0.92) increased more steeply than the titers in 17syn+-infected TG (linear regression; slope = 218 PFU/h; *R*^2^ = 0.87). The slopes of the lines representing the amount of infectious virus produced over time were significantly different between the two viral strains (*P* = 0.0117) (Tables 1 and 2). These higher titers were correlated with earlier detection of virus in the explant media of McKrae-infected TG (Table 2). Whether this reflects an increased rate of ongoing primary reactivation, spread within the TG, or both is not known (11). However, it points to a complicating issue when comparing the times to reactivation of two viral strains in explant using the sampling method. The time of detection in the medium will not only be contingent upon the time of viral exit from latency, but also on the number of neurons initially producing infectious virus; the physical location of these neurons in the three-dimensional tissue; and subsequent spread, amplification of reactivated virus, and ultimately escape into the medium. These confounding variables prevent the accurate determination of the time of reactivation using the sampling method.

The direct comparison of two methods for detecting HSV reactivation in explant in this study brings clarity to some conflicting results regarding HSV reactivation outcomes. The relationship between viral replication during acute infection, the establishment of latency, and subsequent reactivation frequency have been well established. It has been shown that reduction of viral replication during acute infection, through therapeutic intervention (15, 27–30), through changes in the inoculation titer (18, 31), or as a result of a mutation in the virus (18, 32–35), reduces the level of latency. The size of the latent pool has been shown to influence reactivation, with higher numbers of latent genomes correlating directly with increased reactivation frequency for a given viral strain (26, 31, 36–39). These well-established relationships have directed vaccine development, and accordingly, strategies that have successfully limited viral acute infection have also been shown to reduce the establishment of latency and the frequency of reactivation (26, 40, 41). However, a recent study did not find a correlation between acute replication, the number of latent genomes, and the time to reactivation (17). In this study, reactivation of several HSV-1 strains was measured by explanting latently infected ganglia and sampling the media from the explanted cultures daily for infectious virus. Although this method has been used in a number of studies to evaluate reactivation competency (i.e., infectious virus recovery) and rate of release into the medium (16, 42, 43), the ability of the method to accurately reveal the time of initial primary reactivation had not previously been examined. The results of our study show that the sampling method used in this most recent report likely did not reflect the time of initial reactivation, and the comparison of multiple viral strains, which would have been subject to variations in the rate of spread/amplification postexplant, could account for the failure of that study to detect a correlation between the level of latency establishment and reactivation.

In conclusion, while medium sampling from explanted cultures has the advantage of monitoring the same ganglia over time, we have found that (i) the timing of HSV reactivation in explant occurs ∼48 to 72 h earlier than can be detected by sampling of the culture media; (ii) as shown previously (11), virus spreads beyond primary reactivation sites by 48 h postexplant; and (iii) the amount of virus detected in culture media significantly underrepresents the amount of infectious virus in the explanted ganglia. Additional direct comparisons between reactivation methods and viral strains are warranted in order to compare and interpret experimental outcomes between studies.

## MATERIALS AND METHODS

### Cells and viruses.

RSC, originally obtained from B. Roizman at the University of Chicago, were maintained in minimal essential medium (MEM) supplemented with 5% newborn calf serum and incubated at 37°C in a 5% CO_2_ incubator. Virus stocks of HSV-1 strain McKrae (originally obtained from S. Wechsler at Mount Cedar Sinai Medical Center Research Institute) and HSV-1 strain 17syn+ (originally obtained from J. H. Subak-Sharpe at the MRC Virology Unit, Glasgow, Scotland) were generated by routine propagation on RSC monolayers. Infected RSC were harvested and frozen and thawed three times, and the titer was determined by serial 10-fold dilution plaque assay on RSC monolayers. Following a 2-h incubation period, the infected monolayers were overlaid with medium containing 1% carboxymethylcellulose and stained with crystal violet 2 to 3 days later. Stocks were aliquoted and stored at −80°C.

### Inoculation of mice.

All procedures involving animals were approved by the Children's Hospital Institutional Animal Care and Use Committee and were in compliance with NIH guidelines. The animals were housed in American Association for Laboratory Animal Care-approved quarters. C57BL/6, male or female as indicated (bred in house; 6 to 8 weeks old), and Swiss Webster male mice (Envigo; 6 to 8 weeks old) were anesthetized by intraperitoneal injection of sodium pentobarbital (50 mg/kg of body weight) prior to inoculation. A 10-μl drop containing the indicated titer of virus was placed onto each scarified corneal surface (44). In this study, infection of Swiss Webster male mice with 2 × 10^5^ PFU of HSV-1 strain 17syn+ resulted in 15% (6/40 mice) mortality. Infection of C57BL/6 male mice with 2 × 10^6^ PFU of HSV-1 strain 17syn+ resulted in 4% (1/23 mice) mortality. Infection of C57BL/6 female mice with 2 × 10^5^ PFU of HSV-1 strain McKrae resulted in 42% (15/36) mortality; females were selected for further study of McKrae, since mortality was even greater (7/8; 88%) in male C57BL/6 mice infected with McKrae.

### Explant reactivation.

Latent HSV was induced to reactivate *in vitro* by aseptically removing the trigeminal ganglia and placing each individual ganglion in 1.5 ml MEM with 5% newborn calf serum in a single well of a 24-well plate in a 5% CO_2_ incubator at 37°C. Samples (100 μl) of the explanted culture media were taken every 24 h and directly plated on RSC monolayers. Following a 2-h absorption period, the cells were overlaid with medium containing 1% carboxymethylcellulose. The plates were stained with crystal violet approximately 2 to 3 days later, and the plaques were counted. At the indicated times postexplant, TG were processed for the detection of infectious virus or immunohistochemical analysis.

### Detection of reactivated virus in TG.

At the indicated times postexplant, TG were removed and homogenized in 1 ml medium as described previously (44). The supernatant was directly plated or serially diluted on RSC monolayers. All samples were absorbed for 2 h and overlaid with medium containing 1% carboxymethylcellulose. The plates were stained with crystal violet approximately 2 to 3 days later, and the plaques were counted. It had been determined previously that this method can detect 50 PFU (31, 45), and more recent reconstitution experiments have been able to detect approximately 10 PFU when ganglia are spiked prior to processing.

### Antibodies and immunohistochemistry.

Cells positive for HSV proteins were detected in whole ganglia as described previously (23). The primary antibody used was polyclonal rabbit anti-HSV (AXL237; Accurate) diluted 1:3,000, and the secondary antibody used was horseradish peroxidase (HRP)-labeled goat anti-rabbit (Vector) diluted 1:500 in PBS containing 2% bovine serum albumin, 5% dimethyl sulfoxide (DMSO), and 5% normal horse serum. Color development was achieved by exposing the ganglia to a 0.1 M Tris (pH 8.2) solution containing 250 μg of diaminobenzidine (Aldrich)/ml and 0.004% H_2_O_2_ for approximately 5 min. All the slides were viewed under an Olympus BX40 microscope and photographed with an AxioCamHRc (Zeiss).

### Statistical analysis.

The indicated statistical analyses were performed using GraphPad Prism software (GraphPad Software, San Diego, CA). A *P* value of < 0.05 was considered significant. The data are reported as means and standard deviations.
